# visGReMLIN: graph mining-based detection and visualization of conserved motifs at 3D protein-ligand interface at the atomic level

**DOI:** 10.1186/s12859-020-3347-7

**Published:** 2020-03-11

**Authors:** Vagner S. Ribeiro, Charles A. Santana, Alexandre V. Fassio, Fabio R. Cerqueira, Carlos H. da Silveira, João P. R. Romanelli, Adriana Patarroyo-Vargas, Maria G. A. Oliveira, Valdete Gonçalves-Almeida, Sandro C. Izidoro, Raquel C. de Melo-Minardi, Sabrina de A. Silveira

**Affiliations:** 1 Department of Computer Science, Universidade Federal de Viçosa, Viçosa, 36570-900 Brazil; 2 Department of Biochemistry and Immunology, Universidade Federal de Minas Gerais, Belo Horizonte, 31270-901 Brazil; 3 Department of Biochemistry and Molecular Biology, Universidade Federal de Viçosa, Viçosa, 36570-900 Brazil; 4 Instituto de Biotecnologia aplicada à Agropecuária (BIOAGRO), Universidade Federal de Viçosa, Viçosa, 36570-900 Brazil; 5 Department of Computer Engineering, Advanced Campus at Itabira, Universidade Federal de Itajubá, Itabira, 35903-087 Brazil; 6 Department of Computer Science, Universidade Federal de Minas Gerais, Belo Horizonte, 31270-901 Brazil; 7 Department of Production Engineering, Universidade Federal Fluminense, Petrópolis, 25650-050 Brazil; 8 European Molecular Biology Laboratory, European Bioinformatics Institute (EMBL-EBI), Hinxton, CB10 1SD UK

## Abstract

**Background:**

Interactions between proteins and non-proteic small molecule ligands play important roles in the biological processes of living systems. Thus, the development of computational methods to support our understanding of the ligand-receptor recognition process is of fundamental importance since these methods are a major step towards ligand prediction, target identification, lead discovery, and more. This article presents visGReMLIN, a web server that couples a graph mining-based strategy to detect motifs at the protein-ligand interface with an interactive platform to visually explore and interpret these motifs in the context of protein-ligand interfaces.

**Results:**

To illustrate the potential of visGReMLIN, we conducted two cases in which our strategy was compared with previous experimentally and computationally determined results. visGReMLIN allowed us to detect patterns previously documented in the literature in a totally visual manner. In addition, we found some motifs that we believe are relevant to protein-ligand interactions in the analyzed datasets.

**Conclusions:**

We aimed to build a visual analytics-oriented web server to detect and visualize common motifs at the protein-ligand interface. visGReMLIN motifs can support users in gaining insights on the key atoms/residues responsible for protein-ligand interactions in a dataset of complexes.

## Introduction

At the molecular level, protein receptors constantly interact with small-molecule ligands, such as metabolites or drugs. A variety of protein functions can be attributed to or regulated by these interactions [1]. Understanding how protein-ligand interactions take place has been the goal of many research studies [2–5], as molecular recognition is pivotal in biological processes, including signal transduction, catalysis and the regulation of biological function, to name a few examples.

Identifying conserved interactions between proteins and ligands that are reused across a protein family is a key factor for understanding molecular recognition processes and can contribute to rational drug design, target identification, lead discovery and ligand prediction. Interface forming residues (IFR) are residues in the molecular interface region between proteins. In accordance with Tuncbag et al. [6], protein structures are more conserved than their sequences, and IFRs are even more conserved than whole protein structures. Therefore, IFR can be an invaluable source of information to support the identification of conserved interactions across a set of complexes. In this paper, we are interested in the interface between proteins and ligands. We consider ligands to be small non-protein molecules. On one hand, proteins can be promiscuous, as they interact with different ligands [7, 8]. On the other hand, ligands can also be promiscuous, such as when one ligand is recognized by different proteins [9]. Thus, it is reasonable to expect that methods used to detect conserved interactions between proteins and ligands should be able to address both protein and ligand promiscuity.

Several methods have been proposed to identify three-dimensional binding motifs. Here, we briefly review some recent works that are representative examples of the diverse techniques that have already been proposed.

Previous solutions for detecting structural binding motifs for a set of diverse proteins and a common ligand involved protein superimposition based on the ligand and subsequent clustering of the conserved residues or atoms interacting with this ligand. The methods developed by Kuttner et al. [10] and Nebel et al. [11] are examples of this kind of solution. These strategies work well for rigid ligands as they result in structural alignments of good quality due to ligand-induced superimposition. In general, classical methods, such as sequence/structural alignments, are not appropriate for conservation detection when proteins have dissimilar sequences and/or structures [12–14].

Gonçalves-Almeida et al. [15] developed a method based on hydrophobic patch centroids to predict cross-inhibition, also known as inhibitor promiscuity, in serine proteases. IFRs were modeled as a graph in which hydrophobic atoms were the nodes and the contacts between them were the edges. Centroids were used to summarize the connected components of this graph, and conserved centroids, termed hydrophobic patches, were used to characterize, detect and predict cross-inhibition.

In a similar manner, Pires et al. [16] used graphs that consider physicochemical properties of atoms and their contacts to represent protein pockets, generating a signature that perceives distance patterns from protein pockets. Each binding site is represented by a feature vector that encodes a cumulative edge count of contact graphs defined for different cut-off distances, which are used as input data for learning algorithms. This signature does not require any ligand information, and it is independent of molecular orientations.

The motifs computed by the methods designed by Gonçalves-Almeida et al. [15] and Pires et al. [16] can be used to identify, compare, classify and even predict binding sites. However, these motifs include only information on the protein side, and they do not represent the non-covalent interactions established between the ligand and the receptor.

Desaphy et al. [17] encoded structural protein-ligand interactions in graphs and simplified this information in a generic fingerprint, which is a vector of 210 integers, to encompass protein-ligand interaction patterns. To generate the fingerprint, each interaction is described by a pseudoatom. Then, all possible pseudoatom triplets are counted within six distance ranges. Finally, the full vector is pruned to keep the most frequent triplets, resulting in the definition of a frame-invariant fingerprint. In addition to the fingerprint, the authors developed two computational methods to align protein-ligand complexes based on their interaction patterns.

Nakadai et al. [18], in turn, introduced a method based on the differences between residues that were superimposed on small molecule inhibitors and those that were not superimposed to identify key residue pairs as potential targets of protein-protein interfaces. This method addresses a set of structures composed by similar proteins in a complex with different ligands and can support the rational design of inhibitors that target these interfaces. To find the superimposed residues, the authors performed structural alignments.

Recently, a method has been proposed to extract three-dimensional binding motifs that capture information on the ligand and its surrounding residues in protein-ligand complexes. Since molecular function is frequently carried out by a limited number of residues that are reused in functionally conserved proteins during evolution, LibME [19] searches for pocket residues (conserved in terms of chemical properties and spatial position) that surround the ligand. Thus, the resulting motifs are composed of only residues. Additionally, the computation of pocket residue positions relative to the ligand avoids ligand-induced alignment of the pockets. This method is specifically used for diverse proteins binding the same or similar ligands.

In this paper, we propose visGReMLIN (visual Graph Mining strategy to infer protein-Ligand INteraction patterns), a user-friendly web server implementation of our GReMLIN method [20], which uses a graph mining-based strategy to detect conserved structural motifs in large-scale datasets of protein-ligand interactions. visGReMLIN is a visual interactive platform to support the detection of trends and exceptions in protein-ligand interactions by domain specialists, allowing them to explore and make sense of the motifs.

To detect common substructures, which are here called patterns or motifs, in protein-ligand interfaces, we devised a graph mining-based strategy that models the interface as bipartite graphs in which nodes represent atoms from the protein or ligand and edges represent non-covalent interactions between atoms. Nodes are labeled according to their physicochemical properties, and edges are labeled according to the type of interatomic interactions and distance criteria. Next, we perform a clustering analysis on these graphs, which is followed by frequent subgraph mining on each cluster to detect motifs.

In addition, we propose a visual interactive platform to explore protein-ligand interactions and their motifs. The input module automatically searches Protein Data Bank (PDB) [21] for similar structures given an entry and a similarity threshold; alternatively, the user can enter a list of PDB entries. Additionally, visGReMLIN allows users to upload their own structural files (files that have not yet been deposited in PDB). visGReMLIN then starts the computation and notifies the user when the analysis is complete. In our visual representations of protein-ligand interfaces, we use color as a pre-attentive attribute that encodes the physicochemical properties of atoms (nodes) and interactions (edges). Hence, users can see, at a glance, general trends and exceptions regarding the properties of atoms and interactions. Moreover, we provide a variety of filters to explore interactions and their motifs and a text search to help users find residues/atoms of interest. After typing the residue/atom in the text box, our tool highlights the corresponding nodes in the visualization. visGReMLIN supports the selection of a specific motif (frequent subgraph), highlighting it in the context of interface graphs. These graphs are depicted as a 2D schematic representation and in a 3D molecular viewer.

visGReMLIN is a large-scale, alignment-free strategy and its results do not depend on molecular orientation. Furthermore, our method does not specifically require a dataset of different proteins with the same/similar ligands or a dataset with the same/similar proteins and different ligands; it can be used for both types of datasets. In other words, visGReMLIN can be used with datasets containing promiscuous proteins or ligands.

We implemented a whole new version of our strategy from scratch, improving unsupervised learning, frequent subgraph mining and the feature vectors that represent protein-ligand interactions. In addition, the visualization tool was reimplemented and new features added, for instance: (i) visGReMLIN takes as input any protein-ligand complex from PDB; (ii) specific ligands in which users are interested can be selected to compute the motifs; (iii) protein-ligand complexes that are not yet deposited in PDB can be uploaded; (iv) each user can store projects executed in his/her account, facilitating the reproducibility of the experiment; and (v) a user can share a project, allowing multiple users to view, explore and collaborate on the same project.

## Methods

In this section, we explain the visGReMLIN workflow. In addition, we describe the experimental design and datasets used to test and evaluate our strategy.

Before starting to use visGReMLIN, the user needs to register and subsequently log in. This login is useful for organizing projects in the same place without the need to bookmark different submissions. Additionally, the user receives results via email once a process finishes.

**Fig. 1:**
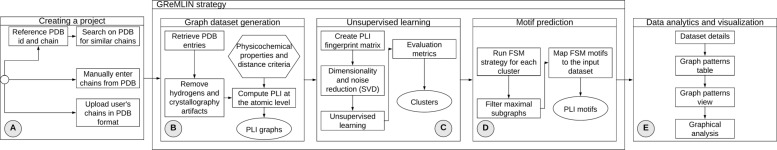
visGReMLIN workflow. The workflow is divided into three main blocks: Creating a project; GReMLIN strategy; and Data analytics and visualization. The white circle represents the starting point; rectangles denote processing steps; ellipsoids represent output files; and hexagons are input files or parameters

Figure 1 shows the visGReMLIN workflow. The web-server has 3 main blocks, which are *Creating a project*, *GReMLIN strategy* and *Data analytics visualization*. Next, we refer to Fig. 1 in order to describe each step performed by visGReMLIN.

### Creating a project

When starting a new project in visGReMLIN, there are 3 options for the user to provide a dataset of protein-ligand complexes (by complex, we mean a PDB id and chain) to be analyzed (Fig.1a):

- Supply a PDB id and chain and let our tool automatically search PDB for similar complexes by selecting an alignment method and an identity percentage;
- Manually enter a dataset of previously selected complexes (type or copy and paste);
- Upload user’s own complexes in PDB format (structures that are not deposited in PDB).

The visGReMLIN *Input module* is shown in Figure 1 of the Supplementary Material. In addition to selecting the input dataset, users can choose the cutoff they want to use for the interaction computation (see Section *Graph dataset generation*), the clustering algorithm and the evaluation metric (see Section *Unsupervised learning*).

### GReMLIN strategy

From now on we use the term GReMLIN to refer to our large-scale, graph-based strategy to detect motifs at the protein-ligand interface. GReMLIN is composed of three main blocks, which we describe in detail next.

#### Graph dataset generation

The first step of GReMLIN is to retrieve the input dataset from PDB according to the information provided in *Creating a project*, which is composed of a set of protein-ligand complexes (Fig.1b).

Then, the structures are processed to remove hydrogen atoms and experimental artifacts. In PDB, structures have been solved using different experimental methods, such as X-ray crystallography, nuclear magnetic resonance (NMR) imaging and neutron diffraction. Although Woińska et al. [22] recently stated that hydrogen atoms can be located by X-ray crystallography, the PDB files of structures solved by NMR or neutron diffraction contain the positions of hydrogens, whereas those from structures solved by X-ray crystallography do not. Thus, to address structures obtained by different experimental methods in a fair manner, we remove hydrogens from PDB complexes before contact computations. Ligands with less than 6 atoms are considered to be experimental artifacts to solve protein structures and are removed. We keep only those complexes that contain at least one ligand.

To compute protein-ligand interactions (PLI), we use a cutoff-dependent approach coupled with a distance criterion. According to previous works [23, 24], a contact is defined between a pair of atoms, (*i*,*j*), if the Euclidean distance between them is less than or equal to a cutoff distance. Thus, we perform contact computations between protein and ligand atoms and based on the physicochemical types of atoms (6 types) and the distance between them, we define the type of interaction established in a similar manner to Silveira et al. [25]; Santana et al. [20] and Fassio et al. [26]. Table 1 of the Additional file 1 presents a list with atom types used in visGReMLIN, which was derived from Fassio et al. [27]. Table 1 provides the standard distance criteria and physicochemical types of atoms involved in each type of interaction. The physicochemical types of atoms from ligands were computed using Pmapper from Chemaxon (http://www.chemaxon.com) at pH 7. It is important to point out that the user is allowed to select his/her own distance criteria, as shown in Figure 1 of the Additional file 1.

**Table 1:** Distance criteria (in Å) and physicochemical types of atoms involved in each type of interaction

Type of interaction	Atom types	Minimum distance	Maximum distance
Aromatic stacking	Two aromatic atoms	1.5	3.5
Hydrogen bond	An acceptor and a donor atom	2.0	3.0
Hydrophobic	Two hydrophobic atoms	2.0	3.8
Repulsive	Two atoms with the same charge	2.0	6.0
Salt bridges	Two atoms with opposite charge	2.0	6.0

**Fig. 2:**
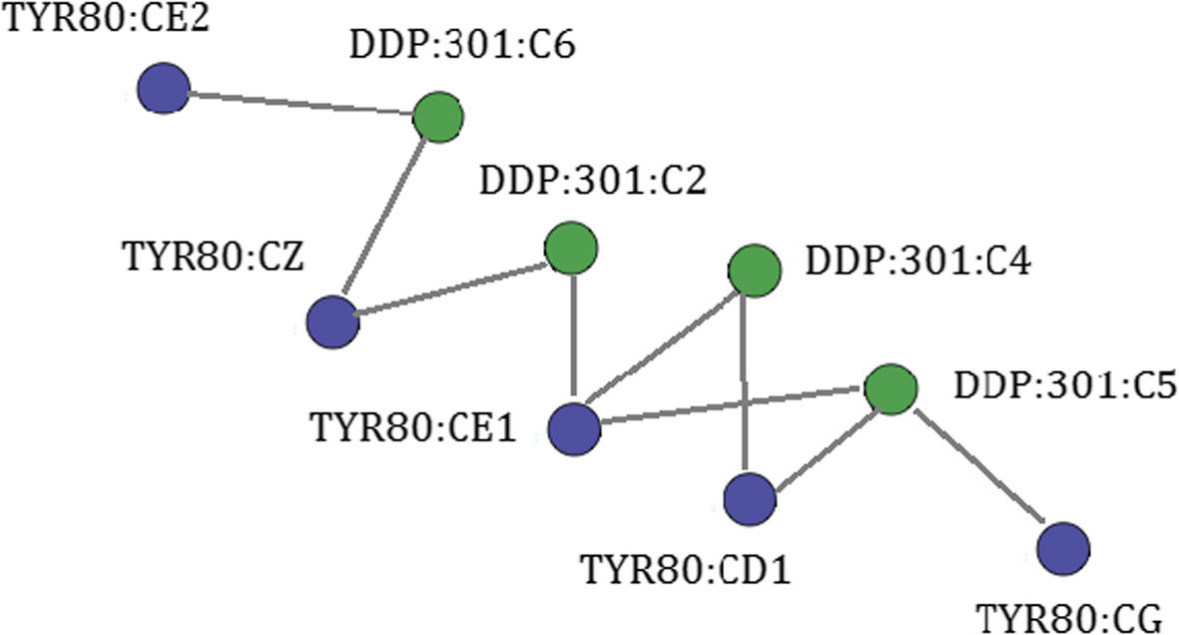
PLI bipartite graph. Scheme of a graph depicting interactions at the interface of ricin protein and its ligand DDP (PDB id 1IL5 chain A). Protein nodes are colored in purple, and ligand nodes are colored in green

In a bipartite graph *G*=(*P*,*L*,*E*), nodes can be divided into two disjoint sets, *P* and *L*, such that every edge in *E* connects a vertex in *P* to one in *L* [28]. We model PLI as bipartite graphs in which nodes depict atoms from a protein (*P*) or ligand (*L*) and edges represent interactions (*E*) among them. Nodes and edges are labeled according to the physicochemical types of atoms and interactions. Figure 2 shows an example of a PLI represented by a bipartite graph. The block *Graph dataset generation* gives these bipartite graphs as output.

#### Unsupervised learning

This block (Figure 1c) takes as input a set of graphs that represent the interfaces between proteins and ligands and segments them in similar groups through an unsupervised learning strategy for motif prediction in the next block.

**Fig. 3:**
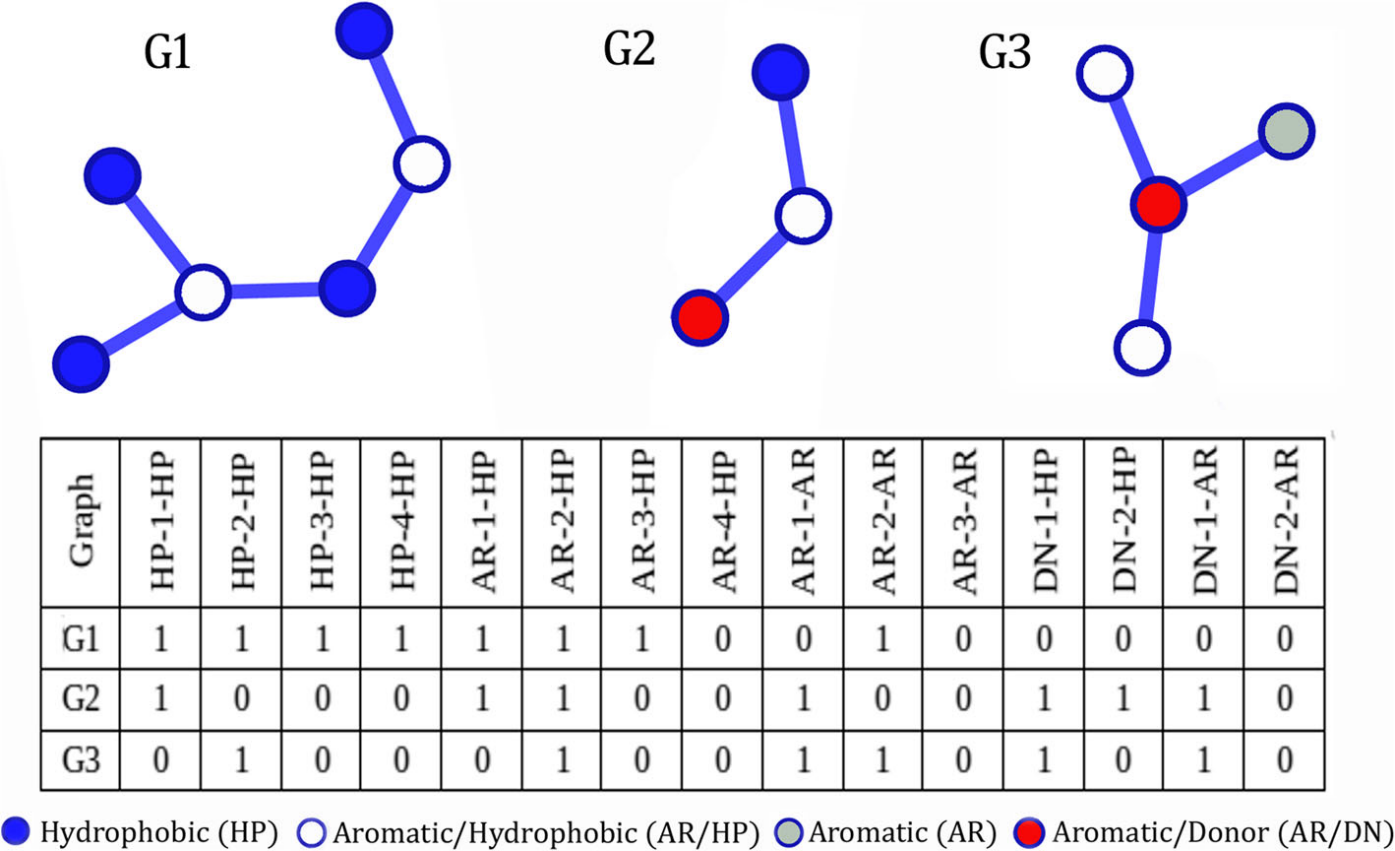
Example of graphs and their feature vectors in the matrix

To summarize our dataset of graphs, we modeled the dataset of PLI graphs as a matrix that contains information from node and edge properties. For each graph, we generate a feature vector in which each position represents the presence of specific properties on a certain graph. Each vector position represents a pair of node properties separated by the edge distance (number of edges between the pair of nodes). To calculate this feature vector, we perform a breadth first search on each graph to obtain each non-cyclic path between all pairs of nodes. These paths are represented by their end nodes with their respective node properties and the distance between them. Figure 3 provides an example of the proposed matrix and an example of a graph and how it is represented by a feature vector in this matrix. For instance, the attribute HP-4-HP means that the graph has two hydrophobic atoms separated by four edges.

To perform dimensionality and noise reduction in the matrix that represents the PLI graphs, we used singular value decomposition (SVD) [29], which is a preprocessing step for the unsupervised learning task. SVD is widely used in data mining for this purpose.

We provide 3 options of clustering algorithms to the users, each of which is based on a different paradigm. K-medoids [30] is centroid based, Agglomerative is hierarchical [31] and Spectral [32] consists of algorithms that cluster data points using eigenvectors of matrices derived from the input data. Additionally, we provide 2 metrics to evaluate the quality of clustering: average silhouette width (asw) [33] and Calinski-Harabaz index [34]. When used with the default parameters, visGReMLIN automatically selects K-medoids and average silhouette width. The output of the Unsupervised learning block is clusters of similar PLI graphs.

#### Motif prediction

The *Motif prediction* block (Fig. 1d) takes as an input the clusters of PLI graphs. Then, a frequent subgraph mining (FSM) experiment is conducted to search for conserved motifs in each cluster.

The algorithm selected for FSM was gSpan [35], which is a highly cited FSM algorithm. In accordance with Jiang et al. [36], considering a graph dataset *D*={*G*_0_,*G*_1_,...,*G*_*n*_}, *support*(*g*) denotes the number of graphs in *D* that have *g* as a subgraph. The purpose of FSM is finding any subgraph *g* whose *support*(*g*)≥*minSup* (a minimum support threshold).

By default, visGReMLIN runs FSM with *support* varying from 0.1 to 1.0 with a step of 0.1 (the amount by which the support varies) for each cluster. As support increases, we obtain subgraphs that are in a high fraction of the graph input dataset. Nonetheless, the number of total subgraphs tends to decrease. Additionally, as the *support* increases, the resulting subgraphs tend to become smaller, which is expected as it is difficult to find bigger patterns in the whole graph input dataset.

Subgraphs resulting from FSM algorithms can be structurally repetitive, as a frequent subgraph can present other frequent subgraphs within it [37]. In accordance with Koyutürk et al. [38], maximal frequent subgraphs are the most interesting ones in many biological networks, as those formed by protein-ligand interfaces. Hence, visGReMLIN filters only the maximal subgraphs. There is no loss of information, as maximal subgraphs contain the discarded graphs.

The output of the FSM algorithm reveals frequent subgraphs and the graphs in the input dataset that they appear in. However, FSM does not provide a direct mapping from each node/edge of frequent subgraphs to the corresponding node/edge in the graphs from the input dataset. This mapping is interesting because it allows users to exactly identify the patterns in the dataset analyzed. To overcome this limitation of FSM, we map the maximal subgraphs to the input graph dataset using the subgraph isomorphism algorithm VF2 [39]. The block *Motif prediction* has as an output the PLI motifs and their mappings to the graph input dataset.

### Data analytics and visualization

*Data analytics and visualization* block (Fig. 1e) is comprised of 4 visualization modules that deliver the results of our strategy in a totally visual and interactive manner, allowing domain specialists to explore and make sense of conserved PLI motifs. visGReMLIN motifs can support users in gaining insights on the key interactions responsible for molecular recognition in a specific dataset. Next, we explain the functionalities of each module.

#### Dataset details

In this module, we present a table that summarizes the unsupervised learning results, as shown in the Additional file 1: Figure 2. The first column shows all the groups, and the second column shows all the complexes of each group. Moreover, column one presents a *graph* icon that displays all the ligands of a specific group and a *network* icon that shows all the input graphs for that group. Regarding the second column, each complex (PDB id and chain) is a link to the structure on the PDB website. This module also has a text search functionality that displays only the lines that contain the entered characters in the table.

#### Graph patterns table

A summary of the PLI motifs is provided in the *Graph patterns table*. As shown in the Additional file 1: Figure 3, by selecting *Grouping columns*, we see a table in which the first column displays the *Motif size* (in number of nodes) segmented by the index of the group, and the second column shows the occurrences of the respective motif segmented by support value. By occurrences, we mean how many motifs were found with a specific size and considering a specific support. By clicking on the column label or on the group or support labels (colored lines inside the table), data can be sorted. A set of filters are offered in the panel Options to explore this table. One can *Filter by group*, *Filter by minimum motif size*, or *Filter by minimum occurrences*. By selecting *Simple table*, we see a heatmap table in which color is a pre-attentive attribute that encodes the frequency of motifs, as provided in the Additional file 1: Figure 4. The darker the shade of blue, the higher the frequency. This table depicts the frequency and size (in number of nodes) of resulting motifs for each group (from unsupervised learning) and for each support value (from FSM). As the choice of support is empirical, we provide a panoramic view of the size, frequency, group and support, to domain specialists to help them decide which support value is appropriate to generate relevant and interesting motifs. There is a compromise between large and frequent motifs. The larger the motif, the smaller its frequency.

#### Motif view

**Fig. 4:**
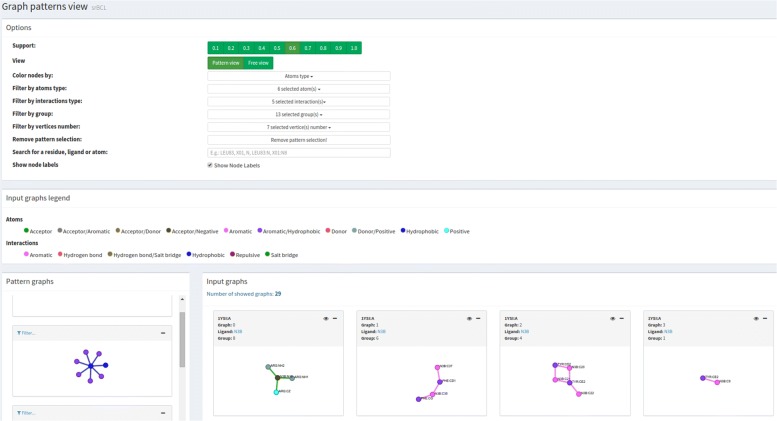
Motif view. This module has 4 main panels: *Options* provides a set of filters to explore PLI motifs; *Graphs legend* displays the colors used to depict atoms and edges; *Motif graphs* shows the set of PLI motifs according to selected filters; and *Input PLI graphs* depicts the PLI graphs that represent the interface between a ligand and a protein

Users can visually explore and make sense of PLI motifs through analytical interaction and navigation in the *Motif view* module, as presented in Figure 4. Motifs can be analyzed alone or in the context of the protein structure, which means that for given a motif, visGReMLIN highlights it in the graphs where it appears. Additionally, the tool presents motifs in schematic 2D representations as graphs or in the context of protein structures in a 3D molecular viewer. This module has 4 main panels that we describe in detail next.

**Options:** provides a variety of filters to interact with the motifs. The common usage is selecting a support value and exploring motifs using the filters below:

- View: displays PLI graphs without segmenting them by choosing *Free view* or shows the PLI graphs in boxes containing PDB id, graph index, ligand name and group index by selecting *Pattern viewFree view*.
- Color nodes by: nodes can be colored according to the atom type or molecule to which they belong (blue for protein and red for ligands).
- Filter by atom type: atoms of the selected type are highlighted (possible types are acceptor, aromatic, donor, hydrophobic, negative and positive).
- Filter by interaction type: interactions of the selected type are highlighted (possible types are aromatic stacking, hydrogen bond, hydrophobic, repulsive and salt bridge).
- Filter by group: displays only graphs of the selected groups from unsupervised learning.
- Filter by vertex number: shows the graphs that contain the selected number of nodes.
- Remove pattern selection: in case a motif has been selected in the panel *Motif graphs*, this option removes the selection.
- Search for a residue, ligand, or atom: highlights nodes from the graphs that contain the residue/ligand/atom in the text search.
- Show node labels: enables or disables the display of labels for the nodes.

**Graphs legend:** this panel presents the legends. Each atom type is associated with a specific color. The same holds for each interaction type.

**Fig. 5:**
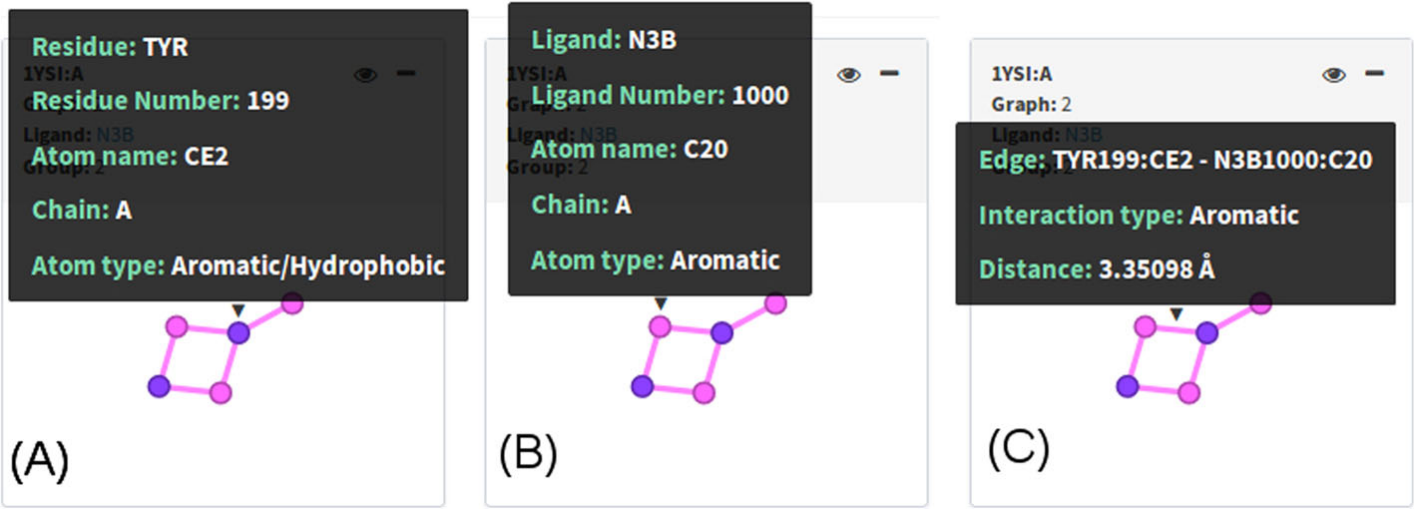
Input PLI graphs. By hovering the mouse over nodes and edges, we see some details on demand. In **a**, we see details of a protein atom, and in **b**, we see details of a ligand atom. Edge details are shown in **c**
- Ligand nodes: ligand name and number, atom name, chain, atom type as shown in Fig. 5b.
- Edges: information about connected atoms, interaction type and distance between these atoms in angstroms (Å). Fig.5c.

**Input PLI graphs:** PLI graphs depicting protein-ligand interfaces for a set of complexes are shown in this panel in accordance with filters from panel *Options* and with the motif chosen in *Motif graphs*. visGReMLIN shows, for each graph, PDB id and chain, graph index, ligand name and group index. By hovering the mouse over the graph, we see some details on demand, depending on the part of the graph:

- Protein nodes: residue name and number, atom name, chain, atom type. An example is provided in Fig.5a

**Motif graphs:** in this panel, users can navigate through motifs, which are the frequent subgraphs from a dataset of protein-ligand complexes represented as graphs. By clicking on a motif, only PLI graphs that contain that motif are displayed on the panel *Input PLI graphs*. Additionally, the motif is highlighted in the context of the PLI graph. Figure 7a provides an example of pattern selection. Types of atoms and interactions are displayed on demand by hovering the mouse over nodes and edges, respectively. Just the motifs from groups selected in *Filter by group* are shown.

To support users in understanding and interpreting the patterns in the context of protein structures, we provide a 3D representation (Fig. 7c) of the protein-ligand interaction graphs in a molecule viewer (which we named *Interaction viewer*) by clicking on the *eye* icon. Additionally, a general 2D visualization of ligands (Fig. 7b) is provided by clicking on the ligand name in any graph from the subsection *Input PLI graphs*. Only ligands from graphs displayed in subsection *Input PLI graphs* are shown in the set of ligands, and the ligand from the graph that the user clicked on is highlighted in green.

#### Graphical analysis

visGReMLIN delivers an interactive interface to show a quantitative summary of motifs extracted from PLI graphs, as shown in Figure 5 of the Additional file 1. The common workflow in this panel is selecting the tab *Atoms type* or *Interactions type* and then choosing a physicochemical type of atoms or interactions to be displayed as histograms. In addition, histogram bars can be organized by *Support* value used in FSM or by *Group* from unsupervised learning.

## Results and discussion

Here, we illustrate how visGReMLIN supports users in gaining insights on the key factors involved in protein-ligand interactions.

Regarding the input parameters, we used the default values for distance criteria, clustering algorithm (K-medoids) and evaluation metric (silhouette coefficient). The support value selected was 0.6, which means that each motif was detected in at least 60% of the PLI input graphs of each group. To show the generality and real-world applicability of our strategy, we used 2 datasets of protein-ligand complexes with different aims.

1. CDK: adapted from [40], this dataset consists in 73 protein-inhibitor complexes of an identical protein with various ligands. This dataset is used to illustrate that our strategy can be used in a scenario involving protein promiscuity.
2. He: comprises 50 complexes involving ATP-binding proteins; thus, it has ATP complexed with various proteins. This dataset was proposed by [19], and it helps us illustrate how our strategy can be used in a scenario involving ligand promiscuity.

The PDB ids of the datasets used in both cases are provided in the Additional file 1: Table 2.

### CDK use case

Using the CDK dataset as an input, we created a project named CDK (available for access on the visGReMLIN website), which resulted in 276 PLI input graphs divided into 14 groups, as shown in the Additional file 1: Figure 6. Here, we present a qualitative analysis in which visGReMLIN motifs are compared to experimentally determined binding site residues/atoms of CDK interacting with the 2 most potent sulfonamide analogue inhibitors obtained in Schonbrunn et al. [40]. Residues/atoms determined as relevant in the PLI in the mentioned work do not represent interactions between CDK and all its possible ligands in our dataset of CDK complexes. Thus, it is expected that the motifs found by visGReMLIN will not contain residues/atoms identical to those that were experimentally determined. Even so, we believe that the comparison is interesting to show that our strategy is able to detect the majority of relevant residues/atoms determined in Schonbrunn et al. [40].

**Fig. 6:**
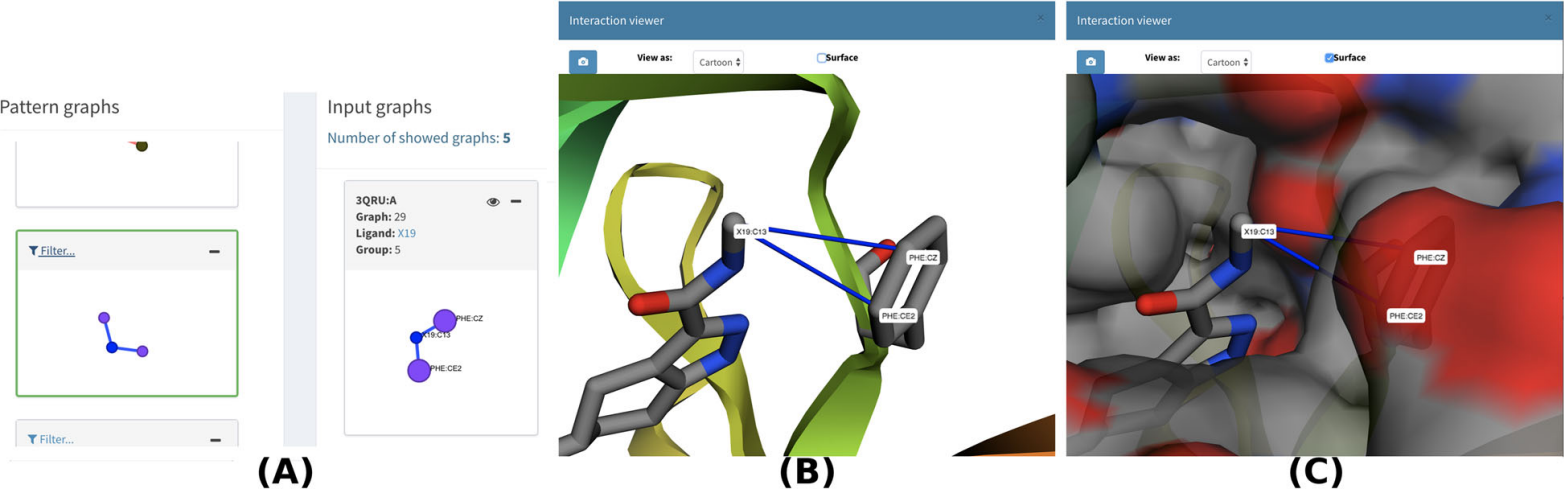
Atoms from the hinge region (PHE82:CE2 and PHE82:CZ) in a visGReMLIN motif. **a** depicts the motif (on the left-hand side) and one of the PLI input graphs that contains the motif (on the right-hand side). **b** shows the motif in the context of CDK structure, and **c** displays exact the same structure in the same position as (**b**) but now visualized as a surface in *Interaction viewer*

In Table 2, we show 26 relevant residues/atoms from the CDK binding site. By searching each of these residues/atoms in visGReMLIN motifs, we were able to find 18, which represents approximately 69%. Residue GLN85 was not considered here as it is involved in a water-mediated interaction with the ligand and our strategy does not consider this type of interaction. Residues in bold in Table 2 (LEU83; PHE 82; and GLU81) are well-known relevant residues for CDK as they are in the hinge region of this protein. It is important to point out that our strategy was able to detect all residues/atoms from the CDK hinge region. In Fig. 6, we show an example of atoms from the hinge region detected in a visGReMLIN motif (PHE82:CE2 and PHE82:CZ).

**Fig. 7:**
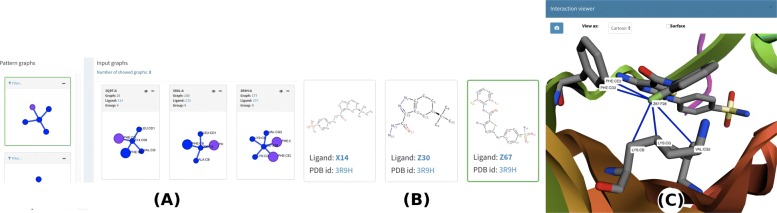
5-node motif from the CDK dataset. The motif is displayed in (**a**) and is highlighted in green on the left-hand side. PLI input graphs from group 9 (where the motif was detected) are shown on the right-hand side of (**a**). **b** provides images of the ligands from the PLI input graphs in group 9 (X14, Z30, and Z67) with ligand Z67 from complex 3R9H.A highlighted in green. **c** shows the motif in complex 3R9H.A in a 3D molecular viewer

**Table 2:** CDK binding site

Residue	Atom	visGReMLIN
ASP145	CB	$ \checkmark $
	CG	×
	OD1	$\checkmark $
LYS33	CB	$\checkmark $
	CD	$\checkmark $
	CE	×
	CG	$\checkmark $
	NZ	∙
ASP86	N	$\checkmark $
	CB	$\checkmark $
	OD1	∙
	OD2	$\checkmark $
LYS89	CB	$\checkmark $
	CE	×
	NZ	∙
HIS84	O	×
**LEU83**	N	$\checkmark $
	O	$\checkmark $
**PHE82**	CE2	$\checkmark $
	CZ	$\checkmark $
**GLU81**	O	$\checkmark $
PHE80	CB	$\checkmark $
	CG	$\checkmark $
	CD2	$\checkmark $
	CE2	$\checkmark $
	CZ	∙

Residues from CDK that interact with the 2 most potent sulfonamide analogue inhibitors

$\checkmark $ Residues/atoms found in patterns;

∙ Found but not in patterns;

× Not found

Next, we present one interesting motif detected by visually inspecting the visGReMLIN results. Figure 7a depicts a motif (highlighted in green on the left-hand side) with 5 nodes, 4 of which (in blue) are hydrophobic atoms and the other (in purple) of which is aromatic/hydrophobic. This motif was found in 3 different complexes (3QRT.A, 3R8L.A, and 3R9H.A) with different ligands (X14, Z30, and Z67) of group 9 (which has 3 PLI graphs). These complexes are shown in Fig. 7a on the right-hand side. Figure 7b displays the 3 different ligands from the PLI input graphs with ligand Z67 from complex 3R9H.A highlighted. Figure 7c presents the motif in the context of the protein structure in a 3D molecular viewer for complex 3R9H.A.

### ATP use case

We created a project called ATP (available for access on visGReMLIN website) in which we use data from [19] as an input. It resulted in 293 PLI graphs segmented in 2 groups, as shown in the Additional file 1: Figure 7. Here, we present a qualitative analysis in which we discuss some interesting motifs detected by visually inspecting the visGReMLIN results.

**Fig. 8:**
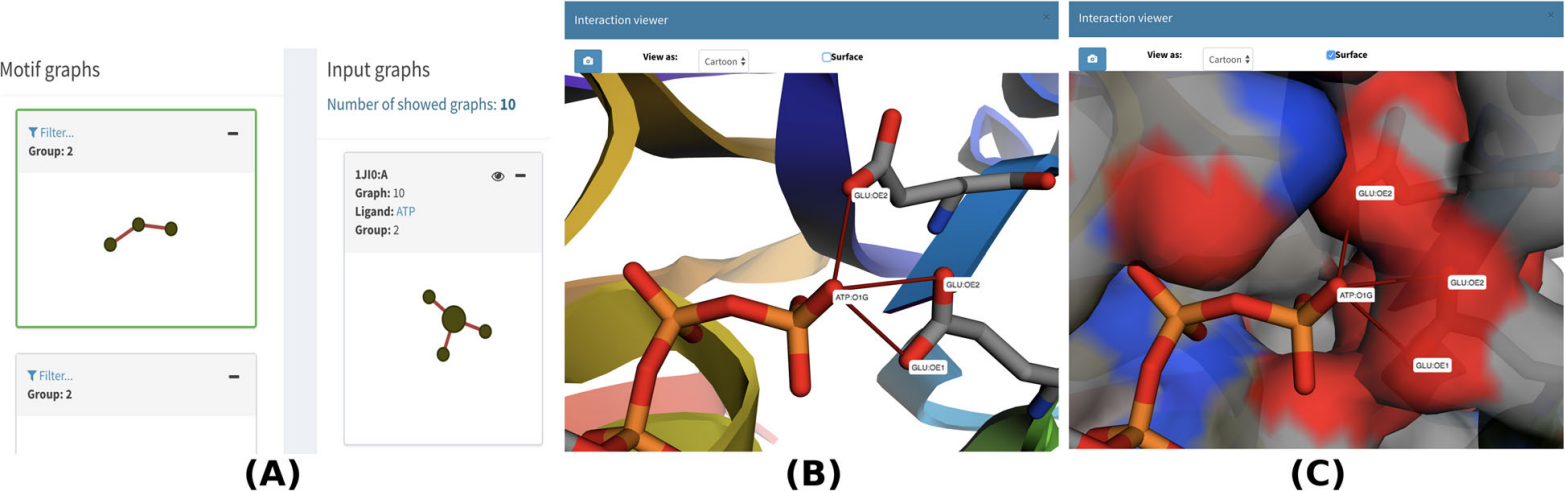
3-node motif from the ATP dataset. The motif is displayed in (**a**) and highlighted in green on the left-hand side. One of the PLI input graphs (1JI0.A) from group 2 in which the motif was detected is shown on the right-hand side of (**a**), and the ATP atom from this motif that interacts most often with the protein, O1G, is shown in a larger size. (**b**) shows the motif in the protein structure. (**c**) adds the protein surface to the previous structure

visGReMLIN found 2 motifs; one motif contains three acceptor/negative nodes connected by 2 repulsive interactions and appears in 17 input graphs. The ATP atom from this motif that interacts most often with the protein is O1G (20 interactions in a total of 10 input graphs), followed by O2B (14 interactions in a total of 8 input graphs) and O1A (11 interactions in a total of 8 input graphs). Figure 8a shows this 3-node motif highlighted in green on the left-hand side and the mapping of such motif to input graph 1JI0.A, which contains O1G interacting with protein atoms, on the right-hand side. In Fig. 8b, we observe this motif in the context of the protein structure, and in Fig. 8c, we add the surface to the molecular view. In the Additional file 1: Table 3, we present the frequency at which each ATP atom of this motif interacts with protein atoms.

**Fig. 9:**
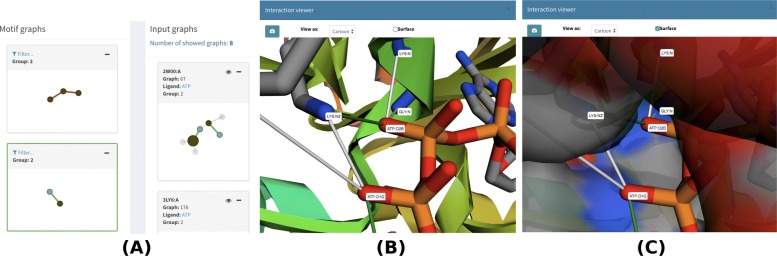
2-node motif from the ATP dataset. **a** displays the motif highlighted in green on the left-hand side. One of the PLI input graphs (2W00.A) from group 2 in which the motif was detected is shown on the right-hand side of (**a**), and the ATP atom from this motif, O2B, is shown in a larger size. **b** shows this motif in the protein structure. (**c**) adds the protein surface to the previous structure

The other motif for ATP consists of two nodes, one donor/positive and the other acceptor/negative, which are connected by a salt bridge and were found in 19 input graphs. In this motif, the ATP atom that interacts most often with the protein is O1G (18 interactions in a total of 12 input graphs), followed by O1B (9 interactions in a total of 8 input graphs) and O2G, O2B and O1A (all establish 8 interactions). Fig. 9a shows this 2-node motif highlighted in green on the left-hand side, and the mapping of such motif to input graph 2W00.A, which contains O2B interacting with protein atoms, on the right-hand side. In Fig. 9b, we observe this motif in the protein structure, and in Fig. 9c, we add the surface to the molecular view.

Of the 9 residues that He et al. [19] computationally calculate as the motif for the ATP dataset (3FVQ.THR4, 2R6G.GLY39, 3EPS.VAL334, 1UA2.GLY19, 4DIN.THR51, 3MN7.TYR306, 2W00.LEU315, 4J7C.ALA57, and 3EPS.LYS461), visGReMLIN was able to detect 1 (LYS) using its default parameters. It was expected that the visGReMLIN motifs would differ from the mentioned work as they are computed in a totally different manner and with different purposes. While He et al. [19] is focused on finding a unique motif (composed of residues) that summarizes the binding for a specific dataset (no matter the size of the dataset), our strategy aims at delivering frequent substructures (common arrangements of atoms) in PLI.

## Conclusion

In this paper, we present visGReMLIN, a user-friendly web-server that brings together a computational strategy to detect motifs at the protein-ligand interface and a visual interactive platform to explore and interpret such patterns. By motifs, we mean frequent subgraphs detected at the interfaces between proteins and ligands. visGReMLIN motifs can support users in gaining insights on the key atoms/residues responsible for protein-ligand interactions in a dataset of complexes.

To illustrate the ability of our strategy to support users in the detection and understanding of motifs, we conducted 2 use cases. In the first one, we used a dataset of 73 identical CDKs in a complex with a varied set of ligands. We compared our motifs to experimental results to show that visGReMLIN is able to find relevant atoms/residues that have been experimentally determined. In the second use case, we used a dataset of 50 complexes that involve the ATP ligand in a complex with different proteins. We performed a qualitative analysis to illustrate the visGReMLIN motifs for ATP, and we also compared our motifs to those found in a computational study. visGReMLIN found approximately 10% of these patterns. We believe that the visGReMLIN motifs differ because while the previous study aimed to find a unique motif composed of residues that summarize the binding for a specific dataset regardless of its size, visGReMLIN focuses on finding frequent structural arrangements of atoms (from the protein and from the ligand) at the interface between proteins and ligands.

In addition, these use cases show that visGReMLIN can be used with a dataset of the same/similar proteins in complex with different ligands and with a dataset of the same/similar ligands in a complex with different proteins, which means that the tool can be used in scenarios involving protein promiscuity and ligand promiscuity. We believe that this functionality is an important result of our work, as many available methods are limited to one of these scenarios.

As future work, we would like to investigate whether the motifs detected by visGReMLIN can be used to predict protein-ligand interactions. Considering that our strategy is able to characterize the interface motifs between a dataset of proteins and ligands, we are interested in using these motifs to help us to choose potential ligands for a specific protein. Additionally, we plan to systematically measure user insights and impressions about motif detection and the proposed visualization to help us improve visGReMLIN.

## Supplementary information

**Additional file 1** Additional figures and tables.

## Data Availability

All the data used in the experiments can be found in the PDB website. visGReMLIN can be accessed by the URL: http://vagner.dti.ufv.br/visgremlin4.

## Abbreviations

ATP: Adenosine triphosphate
CDK: Cyclin-dependent kinase
FSM: Frequent subgraph mining
IFR: Interface forming residues
NMR: Nuclear magnetic resonance
PDB: Protein data bank
PLI: Protein-ligand interactions
