# Supplementary material for: visGReMLIN: graph mining-based detection and visualization of conserved motifs at 3D protein-ligand interface at the atomic level
Source: BMC Bioinformatics. 2020 Mar 11;21(Suppl 2):80. doi: 10.1186/s12859-020-3347-7 (PMC7068867; doi:10.1186/s12859-020-3347-7)
Supplement: Supplementary file 1 — Additional file 1 Additional figures and tables. [file 12859_2020_3347_MOESM1_ESM.pdf]

VISGREMLIN: A GRAPH MINING BASED DETECTION AND VISUALIZATION OF CONSERVED MOTIFS IN 3D PROTEIN-LIGAND INTERFACE AT THE ATOMIC LEVEL - SUPPLEMENTARY MATERIAL

1 METHODS

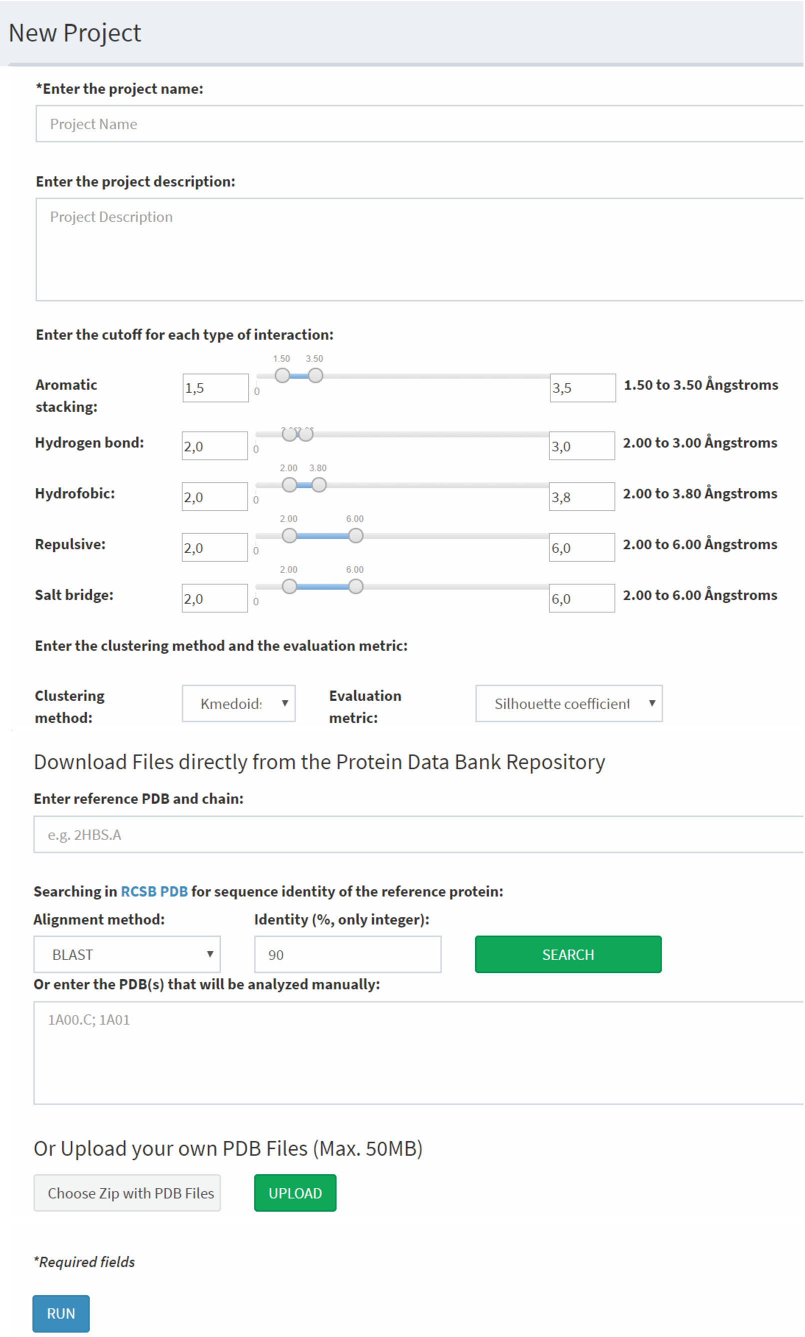

Fig. 1. Starting a new project in visGReMLIN. There are 3 options for the user to provide the dataset of protein-ligand complexes similar to the reference PDB id to be analyzed: **a)** Let our tool to automatically search on PDB for these complexes, by selecting an alignment method and an identity percentage; **b)** Enter a dataset of previously selected complexes manually (type or copy and paste); **c)** Upload users own complexes in PDB format (structures that are not deposited in PDB).

Table 1: Atom types used in visGReMLIN.

Molecule	Atom name	Hydrophobic	Aromatic	Positive	Negative	Donor	Acceptor
ALA	N					X	
ALA	CA						
ALA	C						
ALA	O						X
ALA	CB	X					
ARG	N					X	
ARG	CA						
ARG	C						
ARG	O						X
ARG	CB	X					
ARG	CG	X					
ARG	CD						
ARG	NE			X		X	
ARG	CZ			X			
ARG	NH1			X		X	
ARG	NH2			X		X	
ASN	N					X	
ASN	CA						
ASN	C						
ASN	O						X
ASN	CB	X					
ASN	CG						
ASN	OD1						X
ASN	ND2					X	
ASP	N					X	
ASP	CA						
ASP	C						
ASP	O						X
ASP	CB	X					
ASP	CG						
ASP	OD1				X		X
ASP	OD2				X		X
CYS	N					X	
CYS	CA						
CYS	C						
CYS	O						X
CYS	CB	X					
CYS	SG					X	X
GLN	N					X	
GLN	CA						
GLN	C						
GLN	O						X
GLN	CB	X					
GLN	CG	X					
GLN	CD						
GLN	OE1						X
GLN	NE2					X	
GLU	N					X	
GLU	CA						
GLU	C						
GLU	O						X
GLU	CB	X					
GLU	CG	X					
GLU	CD						
GLU	OE1				X		X

Table 1 continued from previous page

Molecule	Atom name	Hydrophobic	Aromatic	Positive	Negative	Donor	Acceptor
GLU	OE2				X		X
GLY	N					X	
GLY	CA						
GLY	C						
GLY	O						X
HIS	N					X	
HIS	CA						
HIS	C						
HIS	O						X
HIS	CB	X					
HIS	CG		X				
HIS	ND1		X	X		X	X
HIS	CD2		X				
HIS	CE1		X				
HIS	NE2		X	X		X	X
ILE	N					X	
ILE	CA						
ILE	C						
ILE	O						X
ILE	CB	X					
ILE	CG1	X					
ILE	CG2	X					
ILE	CD1	X					
LEU	N					X	
LEU	CA						
LEU	C						
LEU	O						X
LEU	CB	X					
LEU	CG	X					
LEU	CD1	X					
LEU	CD2	X					
LYS	N					X	
LYS	CA						
LYS	C						
LYS	O						X
LYS	CB	X					
LYS	CG	X					
LYS	CD	X					
LYS	CE						
LYS	NZ			X		X	
MET	N					X	
MET	CA						
MET	C						
MET	O						X
MET	CB	X					
MET	CG	X					
MET	SD						X
MET	CE	X					
PHE	N					X	
PHE	CA						
PHE	C						
PHE	O						X
PHE	CB	X					
PHE	CG	X	X				
PHE	CD1	X	X				
PHE	CD2	X	X				

Table 1 continued from previous page

Molecule	Atom name	Hydrophobic	Aromatic	Positive	Negative	Donor	Acceptor
PHE	CE1	X	X				
PHE	CE2	X	X				
PHE	CZ	X	X				
PRO	N						
PRO	CA						
PRO	C						
PRO	O						X
PRO	CB	X					
PRO	CG	X					
PRO	CD						
SER	N					X	
SER	CA						
SER	C						
SER	O						X
SER	CB						
SER	OG					X	X
THR	N					X	
THR	CA						
THR	C						
THR	O						X
THR	CB						
THR	OG1					X	X
THR	CG2	X					
TRP	N					X	
TRP	CA						
TRP	C						
TRP	O						X
TRP	CB	X					
TRP	CG	X	X				
TRP	CD1		X				
TRP	CD2	X	X				
TRP	NE1		X			X	
TRP	CE2		X				
TRP	CE3	X	X				
TRP	CZ2	X	X				
TRP	CZ3	X	X				
TRP	CH2	X	X				
TYR	N					X	
TYR	CA						
TYR	C						
TYR	O						X
TYR	CB	X					
TYR	CG	X	X				
TYR	CD1	X	X				
TYR	CD2	X	X				
TYR	CE1	X	X				
TYR	CE2	X	X				
TYR	CZ		X				
TYR	OH					X	X
VAL	N					X	
VAL	CA						
VAL	C						
VAL	O						X
VAL	CB	X					
VAL	CG1	X					
VAL	CG2	X					

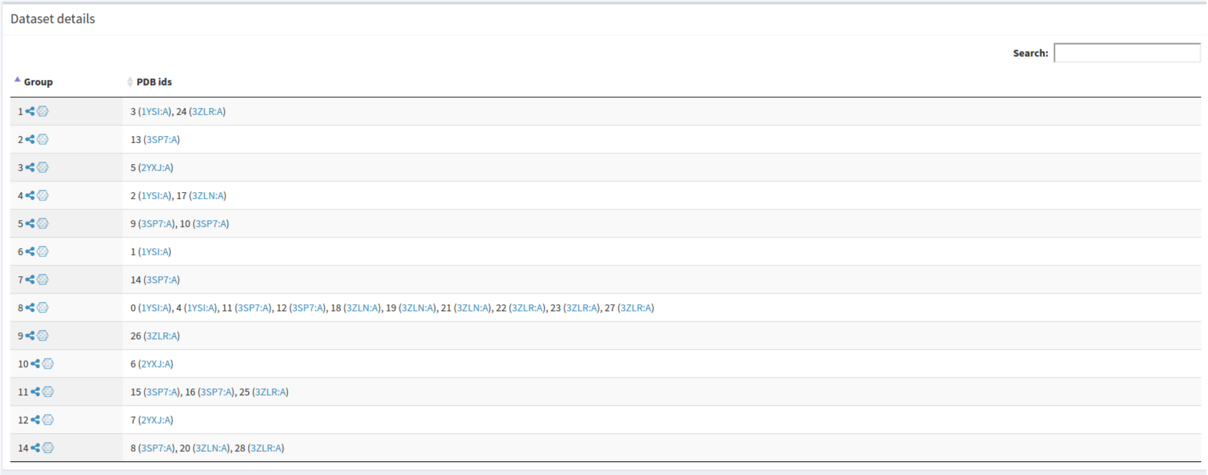

Dataset details

Search:

Group	PDB ids
1	3 (1YSI:A), 24 (3ZLR:A)
2	13 (3SP7:A)
3	5 (2YXJ:A)
4	2 (1YSI:A), 17 (3ZLN:A)
5	9 (3SP7:A), 10 (3SP7:A)
6	1 (1YSI:A)
7	14 (3SP7:A)
8	0 (1YSI:A), 4 (1YSI:A), 11 (3SP7:A), 12 (3SP7:A), 18 (3ZLN:A), 19 (3ZLN:A), 21 (3ZLN:A), 22 (3ZLR:A), 23 (3ZLR:A), 27 (3ZLR:A)
9	26 (3ZLR:A)
10	6 (2YXJ:A)
11	15 (3SP7:A), 16 (3SP7:A), 25 (3ZLR:A)
12	7 (2YXJ:A)
14	8 (3SP7:A), 20 (3ZLN:A), 28 (3ZLR:A)

Fig. 2. The Dataset Details section shows the results of the clustering of the connected graphs that represent the interactions between a given protein and ligand.

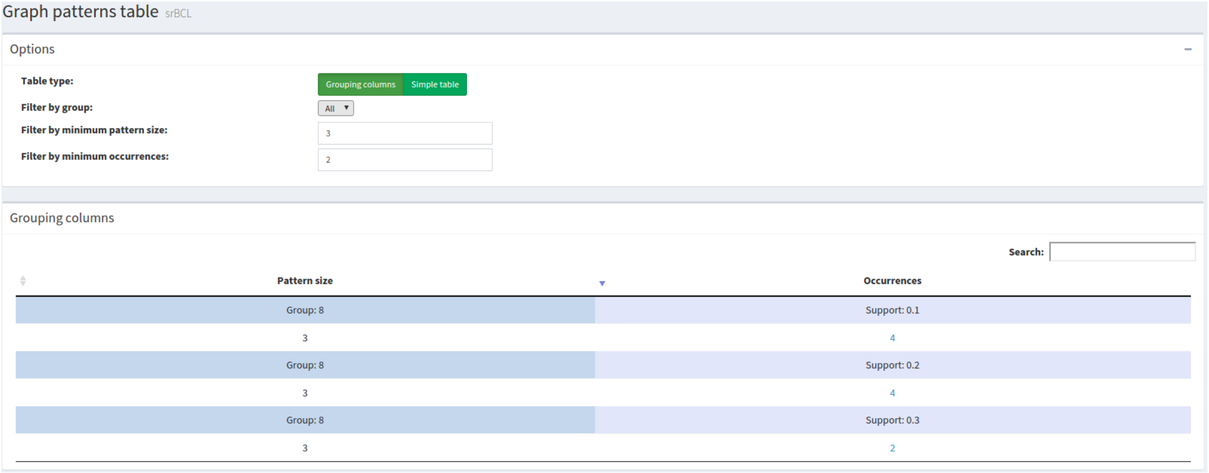

Graph patterns table srBCL

Options

Table type: Grouping columns | Simple table

Filter by group: All

Filter by minimum pattern size: 3

Filter by minimum occurrences: 2

Grouping columns

Search:

Pattern size	Occurrences
Group: 8	Support: 0.1
3	4
Group: 8	Support: 0.2
3	4
Group: 8	Support: 0.3
3	2

Fig. 3. The Graph patterns table page shows a summary of the patterns found for each group and support.

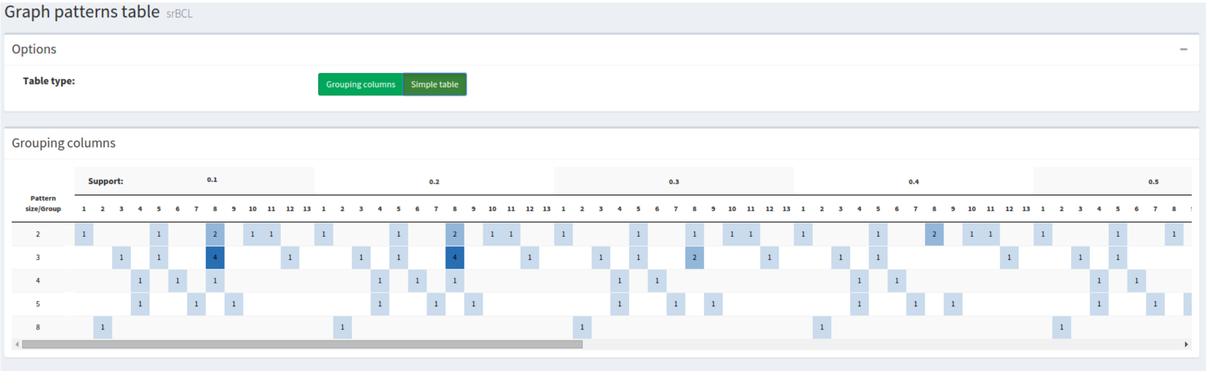

Fig. 4. The simple table shows the number of times that a pattern of a given group, support and size appears.

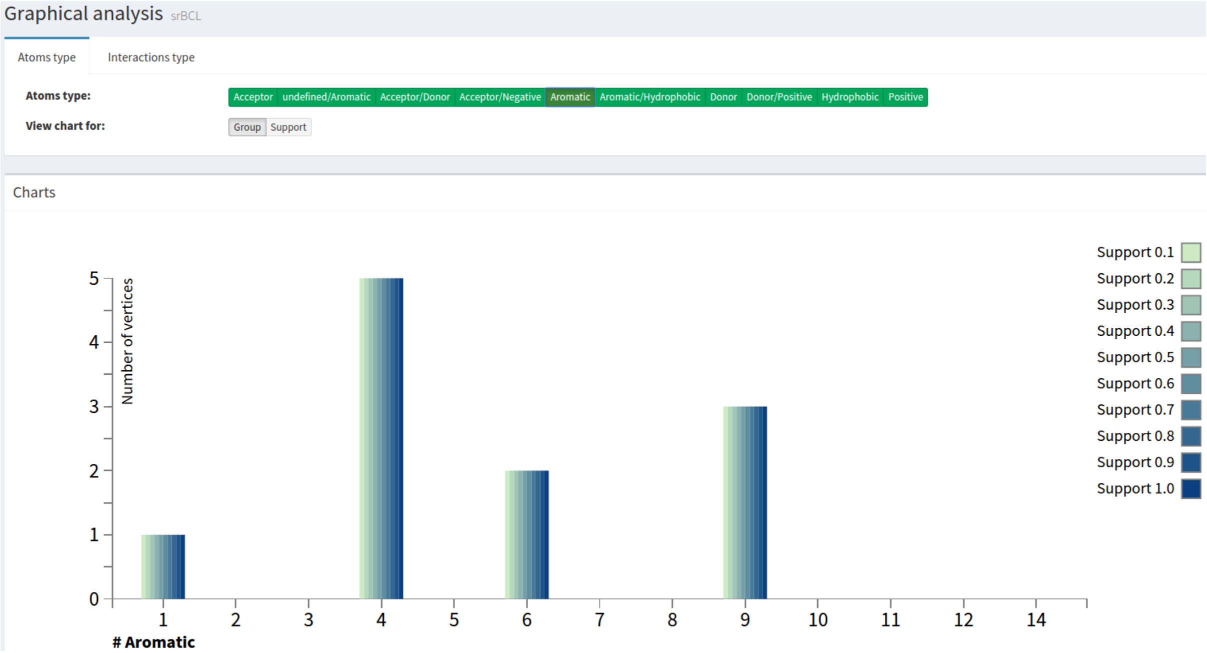

Fig. 5. Summary of motifs extracted from PLI graphs.

2 RESULTS AND DISCUSSION

Group	PDB ids
0	0 (3QL8:A), 3 (3QQF:A), 6 (3QQG:A), 8 (3QQH:A), 10 (3QQJ:A), 12 (3QQK:A), 14 (3QQK:A), 33 (3QTQ:A), 36 (3QTQ:A), 37 (3QTR:A), 39 (3QTR:A), 41 (3QTS:A), 42 (3QTS:A), 45 (3QTU:A), 47 (3QTU:A), 48 (3QTW:A), 50 (3QTW:A), 52 (3QTX:A), 53 (3QTX:A), 55 (3QTZ:A), 56 (3QTZ:A), 57 (3QU0:A), 58 (3QU0:A), 61 (3QWJ:A), 67 (3QWK:A), 73 (3QX2:A), 77 (3QX4:A), 80 (3QXO:A), 84 (3QXP:A), 85 (3QXP:A), 86 (3QZF:A), 88 (3QZF:A), 91 (3QZG:A), 99 (3QZI:A), 107 (3R1S:A), 109 (3R1Y:A), 116 (3R28:A), 121 (3R6X:A), 124 (3R71:A), 127 (3R73:A), 130 (3R7E:A), 139 (3R7U:A), 142 (3R7V:A), 145 (3R7Y:A), 161 (3R8U:A), 163 (3R8U:A), 166 (3R8V:A), 168 (3R8V:A), 170 (3R8Z:A), 171 (3R8Z:A), 175 (3R9D:A), 176 (3R9D:A), 178 (3R9H:A), 179 (3R9H:A), 184 (3R9N:A), 185 (3R9N:A), 187 (3R9O:A), 188 (3R9O:A), 193 (3RAH:A), 194 (3RAH:A), 199 (3RAI:A), 204 (3RAK:A), 205 (3RAK:A), 207 (3RAL:A), 208 (3RAL:A), 210 (3RJC:A), 211 (3RJC:A), 212 (3RK5:A), 214 (3RK5:A), 215 (3RK7:A), 216 (3RK7:A), 217 (3RK9:A), 219 (3RK9:A), 221 (3RKB:A), 222 (3RKB:A), 231 (3RMF:A), 232 (3RMF:A), 233 (3RNI:A), 234 (3RNI:A), 242 (3RPO:A), 246 (3RPR:A), 248 (3RPR:A), 250 (3RPV:A), 252 (3RPV:A), 254 (3RPV:A), 255 (3RPY:A), 256 (3RPY:A), 259 (3RZB:A), 260 (3RZB:A), 263 (3S00:A), 265 (3S00:A), 266 (3S0O:A), 268 (3S0O:A), 270 (3S1H:A), 271 (3S1H:A), 274 (3SQQ:A), 275 (3SQQ:A)
1	2 (3QQF:A), 5 (3QQF:A), 11 (3QQJ:A), 17 (3QQL:A), 22 (3QRT:A), 24 (3QRT:A), 26 (3QRU:A), 40 (3QTS:A), 49 (3QTW:A), 60 (3QWJ:A), 63 (3QWJ:A), 64 (3QWK:A), 66 (3QWK:A), 68 (3QX2:A), 69 (3QX2:A), 72 (3QX2:A), 74 (3QX4:A), 75 (3QX4:A), 79 (3QXO:A), 82 (3QXO:A), 92 (3QZH:A), 96 (3QZH:A), 98 (3QZI:A), 101 (3QZI:A), 102 (3R1Q:A), 103 (3R1Q:A), 105 (3R1S:A), 106 (3R1S:A), 111 (3R1Y:A), 112 (3R28:A), 113 (3R28:A), 118 (3R6X:A), 119 (3R6X:A), 122 (3R71:A), 123 (3R71:A), 128 (3R73:A), 131 (3R7E:A), 132 (3R7I:A), 133 (3R7I:A), 135 (3R7I:A), 137 (3R7U:A), 138 (3R7U:A), 140 (3R7U:A), 141 (3R7V:A), 144 (3R7Y:A), 148 (3R8L:A), 153 (3R8M:A), 157 (3R8P:A), 160 (3R8U:A), 165 (3R8V:A), 182 (3R9N:A), 191 (3RAH:A), 195 (3RAI:A), 197 (3RAI:A), 201 (3RAI:A), 203 (3RAK:A), 206 (3RAL:A), 213 (3RK5:A), 224 (3RM6:A), 225 (3RM6:A), 226 (3RM6:A), 235 (3ROY:A), 237 (3ROY:A), 239 (3RPO:A), 241 (3RPO:A), 244 (3RPR:A), 253 (3RPV:A), 262 (3S00:A), 272 (3S1H:A), 273 (3SQQ:A)
2	149 (3R8L:A), 159 (3R8P:A), 169 (3R8V:A)
3	4 (3QQF:A), 15 (3QQL:A), 62 (3QWJ:A), 65 (3QWK:A), 70 (3QX2:A), 78 (3QX4:A), 81 (3QXO:A), 94 (3QZH:A), 100 (3QZI:A), 104 (3R1Q:A), 108 (3R1S:A), 110 (3R1Y:A), 117 (3R28:A), 120 (3R6X:A), 125 (3R71:A), 126 (3R73:A), 129 (3R7E:A), 134 (3R7I:A), 136 (3R7U:A), 143 (3R7V:A), 146 (3R7Y:A), 147 (3R83:A), 154 (3R8M:A), 173 (3R8Z:A), 200 (3RAI:A), 227 (3RM6:A), 229 (3RM7:A), 236 (3ROY:A), 238 (3ROY:A), 243 (3RPO:A)
4	1 (3QL8:A), 7 (3QQG:A), 9 (3QQH:A), 23 (3QRT:A), 44 (3QTS:A), 46 (3QTU:A), 51 (3QTX:A), 180 (3R9H:A), 209 (3RAL:A), 230 (3RMF:A), 249 (3RPR:A), 251 (3RPV:A), 257 (3RPY:A)
5	29 (3QRU:A), 115 (3R28:A), 183 (3R9N:A), 192 (3RAH:A), 220 (3RKB:A)
6	13 (3QQK:A), 16 (3QQL:A), 18 (3QQL:A), 20 (3QRT:A), 21 (3QRT:A), 27 (3QRU:A), 28 (3QRU:A), 30 (3QRU:A), 31 (3QRU:A), 32 (3QRU:A), 34 (3QTQ:A), 35 (3QTQ:A), 38 (3QTR:A), 43 (3QTS:A), 59 (3QWJ:A), 71 (3QX2:A), 76 (3QX4:A), 83 (3QXO:A), 89 (3QZG:A), 95 (3QZH:A), 97 (3QZI:A), 114 (3R28:A), 151 (3R8L:A), 152 (3R8L:A), 155 (3R8P:A), 156 (3R8P:A), 164 (3R8U:A), 167 (3R8V:A), 172 (3R8Z:A), 181 (3R9N:A), 189 (3R9O:A), 190 (3RAH:A), 196 (3RAI:A), 198 (3RAI:A), 202 (3RAI:A), 218 (3RK9:A), 223 (3RKB:A), 228 (3RM6:A), 240 (3RPO:A), 247 (3RPR:A), 258 (3RZB:A), 264 (3S00:A), 267 (3S0O:A), 269 (3S0O:A)
7	87 (3QZF:A), 90 (3QZG:A), 245 (3RPR:A)
8	162 (3R8U:A), 174 (3R9D:A), 186 (3R9O:A)
9	25 (3QRT:A), 54 (3QTZ:A), 93 (3QZH:A), 150 (3R8L:A), 158 (3R8P:A), 177 (3R9H:A)
10	19 (3QRT:A), 261 (3S00:A)

Fig. 6. Dataset details for CDK use case. CDK use case resulted in 276 PLI input graphs divided in 11 groups.

Group	PDB ids
1	1 (1A0I:A), 2 (1A0I:A), 3 (1A0I:A), 4 (1H3E:A), 5 (1H3E:A), 6 (1H3E:A), 7 (1H3E:A), 9 (1JI0:A), 11 (1JI0:A), 12 (1JI0:A), 13 (1JI0:A), 14 (1JI0:A), 15 (1QHH:A), 16 (1QHH:A), 17 (1QHH:A), 18 (1TF7:A), 19 (1TF7:A), 20 (1TF7:A), 21 (1TF7:A), 23 (1TF7:A), 24 (1TF7:A), 25 (1TF7:A), 26 (1TF7:A), 27 (1TF7:A), 28 (1TF7:A), 29 (1TF7:A), 30 (1UA2:A), 31 (1UA2:A), 32 (1UA2:A), 33 (1UA2:A), 34 (1Z7E:A), 35 (1Z7E:A), 36 (1Z7E:A), 37 (1Z7E:A), 38 (1Z7E:A), 39 (2CG9:A), 40 (2CG9:A), 41 (2CG9:A), 42 (2CG9:A), 43 (2CG9:A), 44 (2HVY:A), 45 (2HVY:A), 46 (2HVY:A), 47 (2J9L:A), 48 (2J9L:A), 49 (2J9L:A), 50 (2J9L:A), 51 (2J9L:A), 52 (2J9L:A), 53 (2JJX:A), 56 (2PBZ:A), 57 (2PBZ:A), 58 (2PBZ:A), 59 (2PBZ:A), 60 (2PBZ:A), 61 (2R6G:A), 62 (2R6G:A), 63 (2R6G:A), 64 (2R6G:A), 65 (2R6G:A), 66 (2R6G:A), 68 (2W00:A), 69 (2W00:A), 70 (2W00:A), 71 (2W00:A), 72 (2W00:A), 74 (2YJE:A), 75 (2YJE:A), 76 (2YJE:A), 77 (2YJE:A), 78 (2YJE:A), 79 (3A8T:A), 80 (3A8T:A), 81 (3A8T:A), 82 (3A8T:A), 83 (3A8T:A), 84 (3A8T:A), 85 (3AMT:A), 86 (3AMT:A), 87 (3AMT:A), 88 (3AMT:A), 89 (3AMT:A), 90 (3AMT:A), 91 (3AMT:A), 93 (3BJU:A), 94 (3BJU:A), 95 (3BJU:A), 96 (3BJU:A), 97 (3BJU:A), 98 (3BJU:A), 99 (3BJU:A), 100 (3D2E:A), 101 (3D2E:A), 102 (3D2E:A), 103 (3D2E:A), 104 (3D2E:A), 105 (3D2E:A), 106 (3D2E:A), 107 (3D2E:A), 108 (3DWL:A), 109 (3DWL:A), 110 (3DWL:A), 111 (3E1Y:A), 113 (3EA0:A), 114 (3EA0:A), 115 (3EA0:A), 116 (3EA0:A), 117 (3EA0:A), 118 (3EA0:A), 119 (3EPS:A), 120 (3EPS:A), 121 (3EPS:A), 122 (3EPS:A), 123 (3EPS:A), 125 (3EPS:A), 126 (3EPS:A), 127 (3FKQ:A), 128 (3FKQ:A), 129 (3FKQ:A), 130 (3FKQ:A), 131 (3FKQ:A), 132 (3FKQ:A), 133 (3FVQ:A), 134 (3FVQ:A), 135 (3FVQ:A), 136 (3FVQ:A), 137 (3FVQ:A), 138 (3FVQ:A), 139 (3H1Q:A), 140 (3H1Q:A), 142 (3H1Q:A), 143 (3H1Q:A), 144 (3H1Q:A), 145 (3HGM:A), 146 (3HGM:A), 147 (3HGM:A), 148 (3HGM:A), 149 (3HGM:A), 150 (3HGM:A), 151 (3HGM:A), 152 (3HGM:A), 153 (3INN:A), 154 (3INN:A), 155 (3INN:A), 156 (3INN:A), 157 (3INN:A), 158 (3INN:A), 159 (3J2T:A), 160 (3J2T:A), 161 (3J2T:A), 162 (3J2T:A), 163 (3J2T:A), 165 (3K5H:A), 166 (3K5H:A), 167 (3K5H:A), 168 (3K5H:A), 169 (3K5H:A), 170 (3K5H:A), 171 (3LKK:A), 173 (3LKK:A), 174 (3LKK:A), 175 (3LKK:A), 177 (3LY6:A), 178 (3LY6:A), 179 (3MN7:A), 180 (3MN7:A), 181 (3MN7:A), 182 (3MN7:A), 183 (3MN7:A), 184 (3MN7:A), 185 (3QB0:A), 186 (3QB0:A), 188 (3QB0:A), 189 (3S3T:A), 190 (3S3T:A), 191 (3S3T:A), 192 (3S3T:A), 193 (3S3T:A), 194 (3S3T:A), 195 (3S3T:A), 196 (3S3T:A), 197 (3S3T:A), 198 (3T54:A), 200 (3T54:A), 201 (3T54:A), 202 (3T54:A), 203 (3T54:A), 204 (3T54:A), 205 (3T54:A), 206 (3T54:A), 208 (3VNQ:A), 209 (3VNQ:A), 210 (3VNQ:A), 211 (3VNQ:A), 212 (3VNQ:A), 213 (3VNQ:A), 214 (3VNQ:A), 215 (3VNQ:A), 216 (3VX4:A), 217 (3VX4:A), 218 (3VX4:A), 219 (3VX4:A), 220 (3VX4:A), 221 (3VX4:A), 223 (3WBZ:A), 224 (3WBZ:A), 226 (3WBZ:A), 227 (3WBZ:A), 228 (3WBZ:A), 229 (3WBZ:A), 231 (3ZC7:A), 232 (3ZC7:A), 233 (3ZC7:A), 234 (3ZC7:A), 235 (3ZC7:A), 236 (3ZC7:A), 237 (3ZCN:A), 238 (3ZCN:A), 239 (3ZCN:A), 240 (3ZCN:A), 241 (3ZCN:A), 242 (3ZCN:A), 243 (3ZCN:A), 244 (3ZIA:A), 245 (3ZIA:A), 246 (3ZIA:A), 247 (3ZIA:A), 248 (3ZIA:A), 250 (4AI6:A), 251 (4AI6:A), 252 (4AI6:A), 253 (4AI6:A), 254 (4B1Z:A), 256 (4B1Z:A), 257 (4B1Z:A), 258 (4B1Z:A), 259 (4BJR:A), 260 (4BJR:A), 262 (4BJR:A), 264 (4DIN:A), 265 (4DIN:A), 266 (4DIN:A), 267 (4DIN:A), 268 (4DIN:A), 269 (4DXL:A), 270 (4DXL:A), 271 (4DXL:A), 272 (4DXL:A), 273 (4DXL:A), 274 (4DXL:A), 275 (4EJ7:A), 277 (4EJ7:A), 278 (4EJ7:A), 279 (4EJ7:A), 280 (4EJ7:A), 281 (4EJ7:A), 282 (4EJ7:A), 283 (4EJ7:A), 284 (4EJ7:A), 285 (4GXQ:A), 286 (4GXQ:A), 287 (4GXQ:A), 288 (4GXQ:A), 289 (4GXQ:A), 290 (4GXQ:A), 291 (4GXQ:A), 292 (4GXQ:A)
2	0 (1A0I:A), 8 (1H3E:A), 10 (1JI0:A), 22 (1TF7:A), 54 (2JJX:A), 55 (2PBZ:A), 67 (2W00:A), 73 (2WPD:A), 92 (3BJU:A), 112 (3EA0:A), 124 (3EPS:A), 141 (3H1Q:A), 164 (3K5H:A), 172 (3LKK:A), 176 (3LY6:A), 187 (3QB0:A), 199 (3T54:A), 207 (3VNQ:A), 222 (3WBZ:A), 225 (3WBZ:A), 230 (3ZC7:A), 249 (4AI6:A), 255 (4B1Z:A), 261 (4BJR:A), 263 (4DIN:A), 276 (4EJ7:A)

Fig. 7. Dataset details for ATP use case. ATP use case resulted in 293 PLI input graphs divided in 2 groups.

Table 2. PDB ids of datasets used - Results and discussion

Datasets	Number of complexes	Complexes
ATP	50	1A0I.A, 4EJ7.A, 2R6G.A, 3ZIA.A, 2CG9.A, 3LKK.A, 3HGM.A, 4GXQ.A, 1TF7.A, 2J9L.A, 2W00.A, 3FKQ.A, 3DWL.A, 3FVQ.A, 3VX4.A, 3T54.A, 3EPS.A, 3D2E.A, 3WBZ.A, 3ZCN.A, 3INN.A, 1UA2.A, 2YJE.A, 4BJR.B, 1H3E.A, 4DXL.A, 3J2T.A, 3MN7.A, 2WPD.A, 1JI0.A, 4J7C.A, 4AI6.A, 2PBZ.A, 3K5H.A, 3H1Q.A, 3VNQ.A, 3A8T.A, 1Z7E.A, 3ZC7.A, 4DIN.A, 2HVY.A, 4B1Z.A, 3QB0.A, 2JJX.A, 3S3T.A, 3BJU.A, 3AMT.A, 3EA0.A, 1QHH.A, 3LY6.A
CDK	73	3QL8.A, 3QQF.A, 3QQG.A, 3QQH.A, 3QQJ.A, 3QQK.A, 3QQL.A, 3QRT.A, 3QRU.A, 3QTQ.A, 3QTR.A, 3QTS.A, 3QTU.A, 3QTW.A, 3QTX.A, 3QTZ.A, 3QU0.A, 3QWJ.A, 3QWK.A, 3QX2.A, 3QX4.A, 3QXO.A, 3QXP.A, 3QZF.A, 3QZG.A, 3QZH.A, 3QZI.A, 3R1Q.A, 3R1S.A, 3R1Y.A, 3R28.A, 3R6X.A, 3R71.A, 3R73.A, 3R7E.A, 3R7I.A, 3R7U.A, 3R7V.A, 3R7Y.A, 3R83.A, 3R8L.A, 3R8M.A, 3R8P.A, 3R8U.A, 3R8V.A, 3R8Z.A, 3R9D.A, 3R9H.A, 3R9N.A, 3R9O.A, 3RAH.A, 3RAI.A, 3RAK.A, 3RAL.A, 3RJC.A, 3RK5.A, 3RK7.A, 3RK9.A, 3RKB.A, 3RM6.A, 3RM7.A, 3RMF.A, 3RNI.A, 3ROY.A, 3RPO.A, 3RPR.A, 3RPV.A, 3RPY.A, 3RZB.A, 3S00.A, 3S0O.A, 3S1H.A, 3SQQ.A

Table 3. Number of interactions established by protein and ligand atoms from ATP dataset - Results and discussion

**3-node-pattern**	
Ligand atom	Number of interactions
O1G	18
O1B	9
O2G	8
O1A	8
O2B	8
O2A	1
Residue atom	Number of interactions
LYS NZ	19
ARG NH2	18
ARG NH1	9
ARG NE	6
**2-node-pattern**	
Ligand atom	Number of interactions
O1G	20
O2B	14
O1A	11
O2G	7
O1B	6
O2A	6
Residue atom	Number of interactions
ASP OD1	20
ASP OD2	18
GLU OE2	13
GLU OE1	12
GLU OD2	1
